# Metastasis Associated in Colorectal Cancer 1 (MACC1) mRNA Expression Is Enhanced in Sporadic Vestibular Schwannoma and Correlates to Deafness

**DOI:** 10.3390/cancers15164089

**Published:** 2023-08-14

**Authors:** Maria Breun, Katharina Flock, Jonas Feldheim, Anja Nattmann, Camelia M. Monoranu, Pia Herrmann, Ralf-Ingo Ernestus, Mario Löhr, Carsten Hagemann, Ulrike Stein

**Affiliations:** 1 Section Experimental Neurosurgery, Department of Neurosurgery, University Hospital Würzburg, 97080 Würzburg, Germanynattmann_a@ukw.de (A.N.);; 2 Division of Clinical Neurooncology, Department of Neurology, University Hospital Essen, 45147 Essen, Germany; 3 Department of Ophthalmology, University Hospital Würzburg, 97080 Würzburg, Germany; 4 Institute of Pathology, Department of Neuropathology, University of Würzburg, 97080 Würzburg, Germany; 5 Experimental and Clinical Research Center, Charité-Universitätsmedizin Berlin and Max-Delbrück Center for Molecular Medicine in the Helmholtz Association, 13125 Berlin, Germany; 6 German Cancer Consortium (DKTK), 69120 Heidelberg, Germany

**Keywords:** vestibular schwannoma, metastasis associated in colorectal cancer 1 (MACC1), pathogenesis, deafness, NF2-related schwannomatosis (NF2), mRNA expression

## Abstract

**Simple Summary:**

Vestibular schwannoma (VS), benign cranial nerve sheath tumors of the vestibulocochlear nerve, lack efficacious systemic therapies, especially if they develop in a *NF2*-related schwannomatosis (NF2) background. They cause hearing loss, tinnitus and balance problems. Metastasis associated in colon cancer 1 (MACC1) is a key driver of metastasis. Although MACC1 expression is associated with highly malignant tumors and VS are considered benign, both are attached to the HGF/MET signaling pathway and *MACC1* is a candidate gene localized at a hearing loss-related gene locus. Therefore, it was investigated whether MACC1 might be involved in VS pathogenesis. Surprisingly, *MACC1* expression was not increased in the more aggressive NF2-associated VS but in sporadic VS. Its expression correlated with deafness of the patients during their clinical course. Thus, these data are a rationale for further investigation of the putative role of MACC1 in VS pathogenesis, especially VS cell invasion and concomitant deafness of patients.

**Abstract:**

Vestibular schwannoma (VS) are benign cranial nerve sheath tumors of the vestibulocochlear nerve. Their incidence is mostly sporadic, but they can also be associated with *NF2*-related schwannomatosis (NF2), a hereditary tumor syndrome. Metastasis associated in colon cancer 1 (MACC1) is known to contribute to angiogenesis, cell growth, invasiveness, cell motility and metastasis of solid malignant cancers. In addition, MACC1 may be associated with nonsyndromic hearing impairment. Therefore, we evaluated whether MACC1 may be involved in the pathogenesis of VS. Sporadic VS, recurrent sporadic VS, NF2-associated VS, recurrent NF2-associated VS and healthy vestibular nerves were analyzed for *MACC1* mRNA and protein expression by quantitative polymerase chain reaction and immunohistochemistry. *MACC1* expression levels were correlated with the patients’ clinical course and symptoms. *MACC1* mRNA expression was significantly higher in sporadic VS compared to NF2-associated VS (*p* < 0.001). The latter expressed similar MACC1 concentrations as healthy vestibular nerves. Recurrent tumors resembled the *MACC1* expression of the primary tumors. *MACC1* mRNA expression was significantly correlated with deafness in sporadic VS patients (*p* = 0.034). Therefore, MACC1 might be a new molecular marker involved in VS pathogenesis.

## 1. Introduction

Metastasis associated in colon cancer 1 (MACC1) is a key driver molecule of metastasis in a multitude of solid cancers [1,2]. It acts as an adapter protein for protein–protein interactions as well as a transcription factor regulating angiogenesis, cell growth, invasiveness, cell motility and metastasis by employing the hepatocyte growth factor (HGF)/mesenchymal-epithelial transition (MET)/MACC1 signaling pathway [2]. In addition, it is a strong prognostic marker, correlating with progression-free survival of patients of more than 20 different solid tumors including, e.g., colon cancer or glioblastoma [1,3,4]. Therefore, *MACC1* is mainly expressed in highly malignant and metastatic tumors, without notable expression in benign tumors [1,2]. Interestingly, *MACC1* was identified as a candidate gene localized at the autosomal recessive non-syndromic hearing impairment locus DFNB90, and therefore suggests its association with hearing loss [5].

Hearing loss is a main symptom in patients with vestibular schwannoma (VS), which are benign solid nerve sheath tumors of the vestibulocochlear nerve [6]. They arise from neoplastic Schwann cells of the vestibular part of the 8th cranial nerve. It is the most common tumor of the cerebellopontine angle and the fourth most common intracranial tumor [7]. Typically, these tumors cause hypoacusis, tinnitus and balance problems [6,7,8,9]. VS mainly occur sporadically and unilaterally with an incidence of 1:100,000 individuals. However, in about five percent of the cases, they arise as a hallmark manifestation of a genetic germline mutation, formerly called neurofibromatosis type 2 and recently renamed to NF2-related schwannomatosis (NF2) [10,11,12,13]. NF2 is a genetic disorder with an incidence of 1 in 25,000 individuals [14]. It is characterized by the loss of the *NF2* gene on chromosome 22, which encodes for the tumor suppressor protein moesin-ezrin-radixin-like protein (merlin) [15,16]. Its loss of function results in the activation of the phosphoinositide 3-kinase (PI3K)/Akt/mammalian target of rapamycin complex 1 (mTORC1) pathway as well as the retrovirus-associated DNA sequences (RAS)/rapidly accelerated fibrosarcoma (RAF)/mitogen extracellular signal-regulated kinase (MEK) pathway, which both lead to cell proliferation and inhibit apoptosis [8,16,17,18]. In addition, the HGF/MET signaling pathway is involved in VS pathogenesis including hearing loss [19,20,21,22,23,24]. Although sporadic and NF2-associated VS are both characterized by merlin loss as the driver mutation [15,16,25], NF2-associated VS growth at an earlier age, are more functionally impairing, more adherent to surrounding structures, and show higher recurrence rates [26]. Therefore, they are the more aggressive type of VS. Although diagnostics and surgery are commonly not a big issue for these tumors, there is no good long lasting curative therapy available for NF2-associated VS [12,15,16]. Bevacizumab has been shown to be of benefit for about one-third of patients with NF2-associated VS [27,28]. Its efficacy and safety are currently assessed in clinical trials, although apparent drug resistance and rebound tumor progression after cessation remain unsolved issues [28,29]. Meanwhile, it is unknown why NF2-associated VS are more aggressive compared to sporadic VS. Thus, the identification of additional factors, which might be involved in VS pathogenesis and could serve as therapeutic targets, is a matter of ongoing research [18].

Since NF2-associated VS appear to be more aggressive compared to sporadic VS [6], we hypothesized that MACC1 might be overexpressed in the former tumors, especially because MACC1 is a regulator of the HGF/MET signaling pathway, which is also a driving force in VS pathogenesis [2,19,20,21,22,23,24]. In addition, the putative involvement of MACC1 in the loss of hearing and hearing impairment as a pivotal characteristic of VS was the rationale to investigate whether MACC1 might be involved in VS pathogenesis.

## 2. Materials and Methods

### 2.1. Tissue Samples and Clinical Data

The study was conducted according to the guidelines of the Declaration of Helsinki and approved by the Institutional Review Board of the University Hospital Würzburg (#145/16). Written informed consent was obtained from all patients for the use of their tissue in this study. All patients were treated in the Neurosurgery Department of the University Hospital Würzburg between 2007 and 2019. Directly after surgical excision, half of each tumor sample was embedded in paraffin for immunohistochemistry; the other half was cryopreserved. We additionally employed paraffin embedded tissue blocks from the neuropathology department for immunohistochemical MACC1 staining to further increase the size of our study cohort. All samples were neuropathologically assessed according to EANO guidelines and WHO criteria [30,31]. Forty-nine tumors were diagnosed as sporadic VS, 5 as NF2-associated VS, and 8 (sporadic VS) and 6 (NF2-associated VS) tumors, respectively, were recurrences. Four normal vestibular nerves were obtained from autopsies within the first 24 h after death. Two glioblastoma samples served as staining controls for MACC1 immunohistochemistry.

Clinical information of the patients was collected retrospectively (Table 1). Hearing function and tumor extension were categorized using the Hannover classification of audiometric results [32,33], whereas tumor growth dynamics were classified by magnetic resonance imaging during a “watch and wait” period before surgery [8,34]. Previously, these methods have already been described in detail [35].

### 2.2. mRNA Extraction and Quantitative RT-PCR (qPCR)

Total RNA was extracted from cryopreserved tissue utilizing the Gene Matrix Universal RNA Purification Kit (Roboklon, Berlin, Germany) and reverse transcribed. Purified RNA samples were stored at −80 °C.

Two-step quantitative real-time PCR was performed in parallel and in duplicate per sample, as described previously [1,3,4]. Briefly, a 136 bp *MACC1* amplicon was produced using the following primers and probes: forward primer 5′-TTC TTT TGA TTC CTC CGG TGA-3′, reverse primer 5′-ACT CTG ATG GGC ATG TGC TG-3′ (BioTEZ, Berlin, Germany), fluorescein isothiocyanate probe 5′-GCA GAC TTC CTC AAG AAA TTC TGG AAG ATC TA-3′, and LCRed640 probe 5′-AGT GTT TCA GAA CTT CTG GAC ATT TTA GAC GA-3′ (TIB MolBiol, Berlin, Germany). For Glucose-6-phosphate dehydrogenase (G6PDH), a 113 bp PCR product was amplified (h-G6PDH Housekeeping Gene Set, Roche Diagnostics, Mannheim, Germany). Calibrator cDNA was derived from SW620 colon cancer cells and used in serial dilutions simultaneously in each run. PCR was performed for 10 min at 95 °C and 45 cycles of 10 s at 95 °C, 30 s at 60 °C, and 4 s at 72 °C, each.

### 2.3. Immunohistochemistry

From formalin-fixed paraffin-embedded blocks of the VS tissue 3 µm sections were cut and stained with anti-MACC1 antibody (HPA020081, Sigma Aldrich, St. Louis, MO, USA) using a 1:750 dilution in dilution buffer (DCS, Jena, Germany) as described [3,36]. Glioblastoma sections served as positive staining controls. MACC1 protein expression was visualized using a polylink secondary antibody and a peroxidase kit (Dako; DCS Innovative Diagnostic Systems, Jena, Germany). For counterstaining, hematoxylin was used. Brown staining was indicative for MACC1 expression and analyzed using a LEICA DMI 3000 B microscope, LEICA DFC450 camera, and LAS V4.5 software (all Leica, Wetzlar, Germany). Five different fields of view were captured from each slide at a magnification of 40× and MACC1 staining intensity measured semi-automatically in Fiji [37,38] by processing an in-house programmed macro (Table 2). This scoring methodology has been utilized and published previously in related projects [36,39]. The optical density (OD) was calculated with $OD=\log_{10}\left(\frac{255}{x}\right)$ with x as median intensity of at least six measured intensities [31].

### 2.4. Statistical Analysis

All statistical computations were performed with SPSS Statistics 23 (IBM, Armonk, NY, USA). Normality was tested using the Shapiro–Wilk test and skewness as well as curtosis evaluated. If normal distribution was rejected, differences were determined using the Kruskal–Wallis test with post hoc Dunn’s test/the Mann–Whitney U test with correction of the significances according to Bonferroni. When normal distribution could be assumed, ANOVA was used to compare differences of expression values, Leven’s test to assess the equality of variances, and Dunnett’s T3 was chosen as post hoc test. As most of the statistical analyses comparing two or multiple groups were based on non-parametric tests, the specific test is only stated in the text when another test, except for Kruskal–Wallis test with post hoc Dunn’s test or Mann–Whitney U-test, was used. *p* < 0.05 was considered to be significant. *p*-values represent the alpha-corrected *p*-values, wherever alpha-correction was performed. Correlation was evaluated using the Spearman’s correlation coefficient. Expression data are presented as boxplots. In these plots the middle line displays the median, the hinges represent the quartiles, and whiskers show extreme values up to 1.5 times the height of the boxplot.

## 3. Results

### 3.1. Patient Cohort

A total of 71 patient samples were assessed for MACC1 expression at the mRNA and protein level. This panel was composed of four normal vestibular nerve tissues, 49 sporadic VS, eight recurrences of sporadic VS, five NF2-associated VS and six recurrences of NF2-associated VS. *MACC1* mRNA could be isolated from all of the normal vestibular nerve samples, 22 sporadic VS, five recurrences of sporadic VS, all NF2-associated VS and five recurrences of NF2-associated VS. The tumor and patient characteristics are summarized in Table 1. We rejected the assumption of normal distribution of the *MACC1* mRNA and protein expression (as determined by qPCR and OD) and therefore performed non-parametric tests to compare the expression between groups (Shapiro–Wilk: *p* < 0.05 each).

### 3.2. MACC1 mRNA and Protein Expression in VS

**Figure 1:**
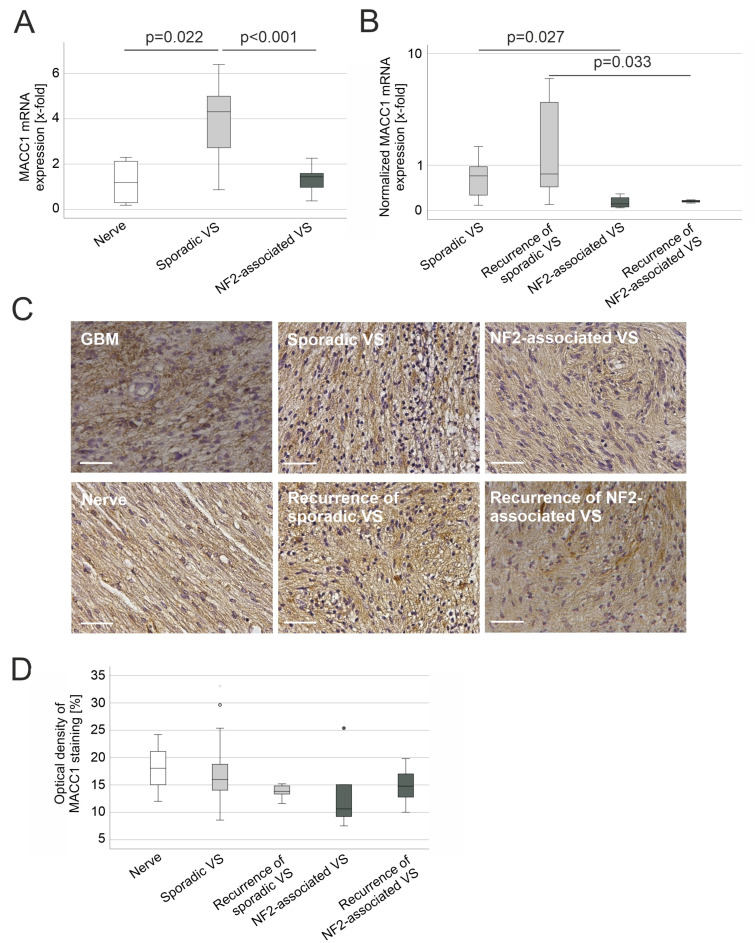
Expression of MACC1 in vestibular schwannoma (VS) specimen. (**A**) Boxplot of *MACC1* mRNA expression in normal vestibular nerve tissue (nerve, n = 4), sporadic VS including recurrences (n = 27) and NF2-related schwannomatosis (NF2)-associated VS including recurrences (n = 10). (**B**) Boxplot of *MACC1* mRNA expression normalized to *G6PDH* expression in sporadic VS (n = 22), recurrence of sporadic VS (n = 5), NF2-associated VS (n = 5) and recurrence of NF2-associated VS (n = 5). (**C**) Representative examples of DAB staining of MACC1 protein expression on paraffin-embedded sections of nerve (n = 3), sporadic VS (n = 49), recurrence of sporadic VS (n = 8), NF2-associated VS (n = 5) and recurrence of NF2-associated VS (n = 6). Glioblastoma (GBM, n = 2) served as positive control. The scale bar indicates 50 µm. (**D**) Quantification of the optical density of MACC1 staining shown in (**D**).

MACC1 mRNA expression of normal vestibular nerve, sporadic VS and NF2-associated VS was analyzed by qPCR. Comparing the absolute MACC1 expression values with each other revealed a mean 3.2-fold higher expression of MACC1 in sporadic VS compared to the normal vestibular nerve (*p* = 0.022) as well as a mean 2.9-fold higher expression compared to NF2-associated VS (*p* < 0.001), whereas there was no difference in MACC1 expression between the latter and normal nerve tissue (Figure 1A). For better comparability, MACC1 mRNA expression was normalized to G6PDH, which is broadly used as a housekeeping gene in VS research [40,41] (Figure S1), and its expression level was compared between sporadic VS, NF2-associated VS and their respective recurrences (Figure 1B). While there was no statistically significant difference in MACC1 mRNA expression between primary tumors and their recurrences, this analysis confirmed the elevated MACC1 mRNA expression in sporadic VS compared to NF2-associated VS (mean 4.7 fold, *p* = 0.027), as well as recurrence of sporadic VS and recurrence of NF2-associated VS (mean 15.8 fold, *p* = 0.033) (Figure 1B).

Immunohistochemistry was performed to confirm MACC1 expression at the protein level (Figure 1C). Although MACC1 protein expression was detected in all analyzed samples, there was no significant difference in the protein expression level between the different VS entities or normal vestibular nerve detectable (Figure 1D).

### 3.3. Correlation of MACC1 mRNA Expression with Clinical Parameters of VS Patients

The normalized MACC1 mRNA expression was enhanced in sporadic VS and their recurrences (sufficient sample size, n = 27), and its expression levels were correlated with the clinical characteristics of VS patients (Table 1). However, the low sample size of NF2-associated VS (n = 5) and of both types of recurrences (both n = 5) would have rendered any correlation analysis unmeaningful. There was no correlation with any of these parameters detectable, including the tumor size as determined by T classification.

**Figure 2:**
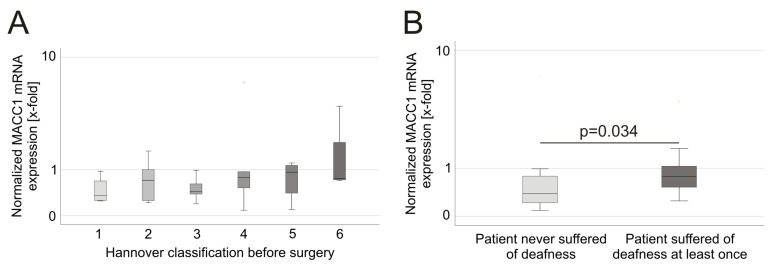
Association of *MACC1* mRNA expression with hearing impairment of patients with sporadic vestibular schwannoma. (**A**) Boxplot of *MACC1* mRNA expression normalized to Glucose-6-phosphate dehydrogenase expression (n = 27) and correlated to the patients’ hearing function according to the Hannover classification of audiometric results [32,33]. A score of H1 refers to pure tone audiometry (PTA) of 0–20 dB and a speech discrimination score (SDS) of 100%–95% (n = 4), H2 to PTA 21–40 dB and SDS 95%–70% (n = 6), H3 to PTA 41–60 dB and SDS 65%–40% (n = 5), H4 to PTA 61–80 dB and SDS 35%–10% (n = 5), H5 to PTA 81–100 dB and SDS 5%–0% (n = 4), and H6 to PTA >100 dB and SDS 0% (n = 3). (**B**) Normalized *MACC1* mRNA expression compared between patients that did (n = 13) or did not (n = 14) experience deafness during their clinical course before surgery.

Remarkably, a closer look at the hearing impairment of patients with sporadic VS, as determined by the Hannover classification of audiometric results [32,33], revealed a tendency of increased MACC1 mRNA expression in the tumors of patients with a score of H6 compared to all other scores (Figure 2A). This score refers to pure tone audiometry (PTA) of >100 dB and a speech discrimination score (SDS) of 0% [32,33]. Therefore, it was analyzed whether MACC1 mRNA expression might be related with deafness in sporadic VS patients during their clinical course before initial surgery. Indeed, MACC1 expression was 1.7-fold higher in the subgroup of patients with sporadic VS suffering from deafness at least once pre-operatively, compared to patients who never suffered from deafness (*p* = 0.034) (Figure 2B).

## 4. Discussion

Patients with NF2-related schwannomatosis develop different types of tumors, e.g., meningiomas, ependymomas or—as a hallmark tumor—VS, due to the loss of the tumor suppressor protein merlin [12,15,16]. NF2-associated VS usually develops bilaterally. Compared to sporadic VS, they grow faster, have a higher recurrence rate, and are more adherent to the cranial nerves and the brain stem [8,17,26,42]. Thus, NF2-associated VS are the more aggressive tumor entity. Compared to sporadic VS they most likely have an additional genetic driver, which cause such increased aggressiveness, since not all of the abovementioned characteristics can be attributed to multifocal tumor growth. However, merlin cannot be the only driver, as it is mutated in both forms of VS [15,16,25]. To the best of our knowledge, the genetic drivers for these differences are not yet known. Therefore, we hypothesized that MACC1 expression might be such a driving component for NF2-associated VS. MACC1 is overexpressed in malignant, especially metastatic tumors, but not in benign or non-metastatic tumor entities [1,2,43]. In addition, it had been shown that it might be involved in hearing impairment [5]. Hence, *MACC1* mRNA expression was analyzed in different entities of VS and it was surprising that *MACC1* was not overexpressed in the more aggressive NF2-associated VS compared to healthy vestibular nerves, but instead in the more benign sporadic VS compared to both.

No correlation of *MACC1* expression with any of the analyzed clinical parameters of the patients were detectable, except for increased MACC1 expression in conjunction with deafness of the patients who suffered from it at least once during their clinical course before surgery. However, due to the low number of samples, the results of the correlation analyses should be interpreted with caution and might be biased. Since tissue samples of VS are rare and even analyses of small cohorts (when compared to solid tumor entities) may yield significant value, we decided to present our observations.

Initially, a hearing impairment caused by VS has not been considered to be genetically determined, but by pressure of the tumor onto the Nervus cochlearis [32]. MACC1 has been shown to be a driver of tumor growth [2,43]. However, there was no correlation of MACC1 expression with VS tumor growth or size. Large tumors may be associated with good hearing function, while some patients with small tumors may suffer from high hearing impairment, suggesting an alternative mechanism besides tumor compression of the nerve [32,44,45,46]. Recently, it has been shown that NLR family pyrin domain containing 3 (NLRP3) inflammasome activation in VS is correlated with VS-induced hearing loss [47,48]. Tumor necrosis factor (TNF) receptors activate NFκB, which induces increased transcription of NLRP3 [49]. Interestingly, both TNFα and NFκB expression are upregulating MACC1 expression in colorectal cancer cells [50]. Although VS are considered benign tumors, they have the potential to invade the surrounding neural tissues. Since *MACC1* has the ability to enhance cell invasiveness [1,2] and it was identified as a candidate gene localized at the autosomal recessive non-syndromic hearing impairment locus DFNB90 mapping to 7p22.1-p15.3, its association with hearing loss was hypothesized [5]. Other genes of the DFNB90 region are ACTB, NXPH1 and PRPS1L1 [5]. Interestingly, these genes have been identified in the transcriptome list of MACC1. Therefore, invasive tumor growth in conjunction with inflammasome activation could be an explanation for an over-proportional hearing loss in some cases with small tumors [35,47,48,51]. Although the evaluation of invasiveness was out of the scope of this study, *MACC1* has been shown to correlate with further invasiveness of tumor cells [2] and therefore might be relevant for hearing impairment caused by tumor invasiveness. This could be elucidated by investigating two cohorts of patients: one group of patients with small VS and poor ability to hear and a second group of patients with similarly sized tumors but good hearing. Expression of *MACC1* and invasiveness of the tumor then could be analyzed to come to a conclusion. However, it will be difficult to collect a sufficient number of samples for this kind of analysis. In addition, MACC1 could be overexpressed and inhibited in VS cell cultures, respectively, and changes in the cells’ invasion as well as proliferation could be measured.

In contrast to sporadic VS, sole surgery is not a long-lasting solution for the treatment of NF2-associated VS, as it is often associated with persistent cranial nerve deficits [12,52]. Bevacizumab might be of benefit for a subgroup of patients, but due to its relevant side effects, e.g., hypertonia or proteinuria, and still pending results from clinical trials, it is only considered for off-label use so far [27,28,29]. Therefore, an efficacious systemic therapy is urgently needed and drug targetable regulatory genes/proteins driving VS development have to be identified [11,15,16,28,52,53]. Two examples of such factors are chemokine receptor-4 (CXCR4) and a disintegrin and metalloproteinase 9 (ADAM9). Their overexpression has been described for VS recently and both should be targetable by specific inhibitors [35,51,54]. Meanwhile, MACC1 is part of the HGF/MET signaling pathway [2], which is also of relevance in VS [20,21,23,24]. HGF/MET signaling has been suggested to play a role in VS-related hearing loss [22] and excessive HGF up- or down-regulation was associated with deafness of patients [19]. The MET inhibitor Crizotinib increases radiosensitivity of VS without adverse effects on hearing and suppresses tumor growth in cell culture experiments, as do other MET inhibitors [21,23,24]. Statins, especially Lovastatin, Fluvastatin and Atorvastatin, have the potential to inhibit MACC1 activity and thereby reduce proliferation and invasion of tumor cells [55,56]. In a recent retrospective investigation of patients with sporadic VS taking statins, the observed reduced growth of the tumors was not statistically significant [57]. However, it is not specified in the publication, which statins have been used. Anyway, NF2-associated VS would be the critical target for such therapies, due to the lack of long lasting curative options. Since *MACC1* was not overexpressed in these tumors, its employment for such a task is unfortunately futile.

## 5. Conclusions

Our pilot study aimed to elucidate whether *MACC1* might be expressed in VS, which would indicate its putative involvement in VS pathogenesis. Therefore, patient-derived tissue samples have been collected for mRNA and protein expression analyses. Although desirable, performing MACC1-inhibitor experiments to show and to confirm MACC1 as a relevant biomarker for sporadic VS and to elucidate its mode of action was out of the scope of this study, but is planned for the future. The data presented here are a rationale for further investigation of a presumable role of MACC1 in sporadic VS pathogenesis, especially VS cell invasion and concomitant deafness of patients.

## Figures and Tables

**Table 1:** Clinical parameters of vestibular schwannoma patients at diagnosis used for correlation analyses.

Patients’ Characteristics	Sporadic VS	NF2-Associated VS
Sex	Female: 24 (42%);	Female:8 (73%);
	Male: 33 (58%)	Male: 3 (27%)
Age at onset of symptoms (median, quartiles)	48.0 (40.0–57.5) years	16.0 (9.5–26.0) years
Age at diagnosis (median, quartiles)	52.0 (41.5–60.0) years	20.0 (14.0–32.0) years
Tumor localization	Solely left nerve: 28 (49%)	Solely left nerve: 1 (9%)
	Solely right nerve: 29 (51%)	Solely right nerve: 2 (18%)
	Bilateral: 0 (0%)	Bilateral: 8 (73%)
Tumor extension	Purely intrameatal (T1): 1 (2%)	Purely intrameatal (T1): 0 (0%)
	Intra- and extrameatal (T2): 7 (12%)	Intra- and extrameatal (T2): 0 (0%)
	Filling the cerebellopontine cistern (T3): 26 (46%)	Filling the cerebellopontine cistern (T3): 2 (18%)
	Brainstem compression ± dislocation of the fourth ventricle (T4): 23 (40%)	Brainstem compression ± dislocation of the fourth ventricle (T4): 9 (82%)
Tumor progress per year ^1^	≤2 mm: 21 (60%);	≤2 mm: 5 (63%);
	≥2 mm: 14 (40%)	≥2 mm: 3 (27%)
Tumor adherence to the brain stem	Yes: 39 (68%); No: 18 (32%)	Yes: 6 (55%); No: 5 (45%)
Antoni classification	Antoni A: 21 (38%)	Antoni A: 4 (44%)
	Antoni B: 6 (11%)	Antoni B: 1 (12%)
	Antoni A/B: 28 (51%)	Antoni A/B: 4 (44%)
Recurrence	8	6 ^2^
Hannover classification of audiometry results	H1: 10 (18%)	H1: 2 (18%)
	H2: 14 (25%)	H2: 0 (0%)
	H3: 8 (14%)	H3: 0 (0%)
	H4: 11 (20%)	H4: 1 (9%)
	H5: 6 (11%)	H5: 0 (0%)
	H6: 7 (12%)	H6: 8 (73%)
House and Brackmann score	1: 51 (89%)	1: 7 (70%)
	2: 5 (9%)	2: 0 (0%)
	3: 1 (2%)	3: 2 (20%)
	4: 0 (0%)	4: 1 (10%)
	5: 0 (0%)	5: 0 (0%)
Primary symptoms	Loss of balance: 19 (33%)	Loss of balance: 2 (18%)
	Loss of hearing: 45 (80%)	Loss of hearing: 6 (55%)
General symptoms	Vertigo: 27 (47%)	Vertigo: 2 (18%)
	Hypoacusis: 49 (86%)	Hypoacusis: 11 (100%)
	Tinnitus: 34 (60%)	Tinnitus: 3 (27%)
	Acute hearing loss ^3^ at least once: 25 (44%)	Acute hearing loss ^3^ at least once: 6 (55%)

^1^ Data of some patients are missing, because surgery was performed directly after diagnosis. ^2^ Prior to recurrence, one VS was irradiated and one received a systemic Avastin therapy. ^3^ Acute hearing loss is defined as the sudden partial or total inability to hear as reported by the patients during anamnesis.

**Table 2:** Macro used for semi-automatic picture evaluation in Fiji [37,38]. Adapted from [36,39].

Macro Commands
input = getDirectory(“Input directory”); output = getDirectory(“Output directory”); Dialog.create(“File type”); Dialog.addString(“File suffix: “, “.tif”, 5); Dialog.show(); suffix = Dialog.getString(); processFolder(input); function processFolder(input) { list = getFileList(input); for (i = 0; i < list.length; i++) { if(File.isDirectory(input + list[i])) processFolder(““ + input + list[i]); if(endsWith(list[i], suffix)) processFile(input, output, list[i]); } } function processFile(input, output, file) { open(input + File.separator + file); name=getTitle(); run(“Colour Deconvolution”, “vectors = [H DAB]”); selectWindow (name+”-(Colour_2)”); run(“Measure”); run(“Close All”); }

## Data Availability

All data are contained within the manuscript. Raw data are available on reasonable request from the corresponding author.

## Supplementary Materials

The following supporting information can be downloaded at: https://www.mdpi.com/article/10.3390/cancers15164089/s1. Figure S1. Box-plot of Glucose-6-phosphate dehydrogenase (G6PDH) mRNA expression in normal vestibular nerve tissue (nerve, n = 4) and vestibular schwannoma (VS) specimen (sporadic VS, n = 22; recurrence of sporadic VS, n = 5; NF2-associated VS, n = 5; recurrence of NF2-associated VS, n = 5). The p-values (ANOVA) refer to the nerve. The different VS-entities were not significantly different from each other, while G6PDH-expression in the normal vestibular nerve was reduced, probably due to increased disintegration in the autopsy material. Therefore, G6PDH was not suitable for comparison analyses of VS with normal vestibular nerve tissue, while it remained an appropriate housekeeping gene to compare VS entities with each other. n.s. = not significant.

## References

[1] Stein U., Walther W., Arlt F., Schwabe H., Smith J., Fichtner I., Birchmeier W., Schlag P.M. (2009). MACC1, a newly identified key regulator of HGF-MET signaling, predicts colon cancer metastasis. Nat. Med..

[2] Radhakrishnan H., Walther W., Zincke F., Kobelt D., Imbastari F., Erdem M., Kortüm B., Dahlmann M., Stein U. (2018). MACC1—The first decade of a key metastasis molecule from gene discovery to clinical translation. Cancer Metastasis Rev..

[3] Hagemann C., Fuchs S., Monoranu C.M., Herrmann P., Smith J., Hohmann T., Grabiec U., Kessler A.F., Dehghani F., Löhr M. (2013). Impact of MACC1 on human malignant glioma progression and patients’ unfavorable prognosis. Neuro-Oncology.

[4] Hagemann C., Neuhaus N., Dahlmann M., Kessler A.F., Kobelt D., Herrmann P., Eyrich M., Freitag B., Linsenmann T., Monoranu C.M. (2019). Circulating MACC1 Transcripts in Glioblastoma Patients Predict Prognosis and Treatment Response. Cancers.

[5] Ali G., Lee K., Andrade P.B., Basit S., Santos-Cortez R.L.P., Chen L., Jelani M., Ansar M., Ahmad W., Leal S.M. (2011). Novel Autosomal Recessive Nonsyndromic Hearing Impairment Locus DFNB90 Maps to 7p22.1-p15.3. Hum. Hered..

[6] Huang X., Xu J., Xu M., Zhou L.-F., Zhang R., Lang L., Xu Q., Zhong P., Chen M., Wang Y. (2013). Clinical features of intracranial vestibular schwannomas. Oncol. Lett..

[7] Carlson M.L., Link M.J. (2021). Vestibular Schwannomas. N. Engl. J. Med..

[8] Asthagiri A.R., Parry D.M., Butman J.A., Kim H.J., Tsilou E.T., Zhuang Z., Lonser R.R. (2009). Neurofibromatosis type 2. Lancet.

[9] Sughrue M.E., Kane A.J., Kaur R., Barry J.J., Rutkowski M.J., Pitts L.H., Cheung S.W., Parsa A.T., Dinh C.T., Bracho O. (2011). A prospective study of hearing preservation in untreated vestibular schwannomas. J. Neurosurg..

[10] Jia H., Lahlou G., Wu H., Sterkers O., Kalamarides M. (2021). Management of Neurofibromatosis Type 2 Associated Vestibular Schwannomas. Curr. Otorhinolaryngol. Rep..

[11] Tan D., Killeen D.E., Kutz J.W. (2021). The Natural History of Vestibular Schwannoma and When to Intervene. Curr. Otorhinolaryngol. Rep..

[12] Sanchez L.D., Bui A., Klesse L.J. (2021). Targeted Therapies for the Neurofibromatoses. Cancers.

[13] Plotkin S.R., Messiaen L., Legius E., Pancza P., Avery R.A., Blakeley J.O., Babovic-Vuksanovic D., Ferner R., Fisher M.J., Friedman J.M. (2022). Updated diagnostic criteria and nomenclature for neurofibromatosis type 2 and schwannomatosis: An international consensus recommendation. Genet. Med..

[14] Evans D.G.R., Moran A., King A., Saeed S., Gurusinghe N., Ramsden R. (2005). Incidence of Vestibular Schwannoma and Neurofibromatosis 2 in the North West of England over a 10-year Period: Higher Incidence than Previously Thought. Otol. Neurotol..

[15] Hanemann C.O. (2008). Magic but treatable? Tumours due to loss of Merlin. Brain.

[16] Lim S.H.-S., Ardern-Holmes S., McCowage G., de Souza P. (2014). Systemic therapy in neurofibromatosis type 2. Cancer Treat. Rev..

[17] Schulz A., Zoch A., Morrison H. (2014). A neuronal function of the tumor suppressor protein merlin. Acta Neuropathol. Commun..

[18] Zhang Y., Long J., Ren J., Huang X., Zhong P., Wang B. (2021). Potential Molecular Biomarkers of Vestibular Schwannoma Growth: Progress and Prospects. Front. Oncol..

[19] Schultz J.M., Khan S.N., Ahmed Z.M., Riazuddin S., Waryah A.M., Chhatre D., Starost M.F., Ploplis B., Buckley S., Velásquez D. (2009). Noncoding Mutations of HGF Are Associated with Nonsyndromic Hearing Loss, DFNB39. Am. J. Hum. Genet..

[20] Torres-Martin M., Lassaletta L., San-Roman-Montero J., DE Campos J.M., Isla A., Gavilan J., Melendez B., Pinto G.R., Burbano R.R., Castresana J.S. (2013). Microarray analysis of gene expression in vestibular schwannomas reveals SPP1/MET signaling pathway and androgen receptor deregulation. Int. J. Oncol..

[21] Dilwali S., Roberts D., Stankovic K.M. (2015). Interplay between VEGF-A and cMET signaling in human vestibular schwannomas and schwann cells. Cancer Biol. Ther..

[22] Blakeley J.O., Ye X., Duda D.G., Halpin C.F., Bergner A.L., Muzikansky A., Merker V.L., Gerstner E.R., Fayad L.M., Ahlawat S. (2016). Efficacy and Biomarker Study of Bevacizumab for Hearing Loss Resulting From Neurofibromatosis Type 2–Associated Vestibular Schwannomas. J. Clin. Oncol..

[23] Fuse M.A., Plati S.K., Burns S.S., Dinh C.T., Bracho O., Yan D., Mittal R., Shen R., Soulakova J.N., Copik A.J. (2017). Combination Therapy with c-Met and Src Inhibitors Induces Caspase-Dependent Apoptosis of Merlin-Deficient Schwann Cells and Suppresses Growth of Schwannoma Cells. Mol. Cancer Ther..

[24] Zhao Y., Liu P., Zhang N., Chen J., Landegger L.D., Wu L., Zhao F., Zhao Y., Zhang Y., Zhang J. (2018). Targeting the cMET pathway augments radiation response without adverse effect on hearing in NF2 schwannoma models. Proc. Natl. Acad. Sci. USA.

[25] Evans D.G.R., Ramsden R.T., Shenton A., Gokhale C., Bowers N.L., Huson S.M., Pichert G., Wallace A. (2007). Mosaicism in neurofibromatosis type 2: An update of risk based on uni/bilaterality of vestibular schwannoma at presentation and sensitive mutation analysis including multiple ligation-dependent probe amplification. J. Med. Genet..

[26] Hexter A., Jones A., Joe H., Heap L., Smith M.J., Wallace A.J., Halliday D., Parry A., Taylor A., Raymond L. (2015). Clinical and molecular predictors of mortality in neurofibromatosis 2: A UK national analysis of 1192 patients. J. Med. Genet..

[27] Shi J., Lu D., Gu R., Sun H., Yu L., Pan R., Zhang Y. (2021). Reliability and toxicity of bevacizumab for neurofibromatosis type 2-related vestibular schwannomas: A systematic review and meta-analysis. Am. J. Otolaryngol..

[28] Tamura R., Toda M. (2022). A Critical Overview of Targeted Therapies for Vestibular Schwannoma. Int. J. Mol. Sci..

[29] Fujii M., Kobayakawa M., Saito K., Inano A., Morita A., Hasegawa M., Mukasa A., Mitsuhara T., Goto T., Yamaguchi S. (2021). Rationale and Design of BeatNF2 Trial: A Clinical Trial to Assess the Efficacy and Safety of Bevacizumab in Patients with Neurofibromatosis Type 2 Related Vestibular Schwannoma. Curr. Oncol..

[30] Goldbrunner R., Weller M., Regis J., Lund-Johansen M., Stavrinou P., Reuss D., Evans D.G., Lefranc F., Sallabanda K., Falini A. (2020). EANO guideline on the diagnosis and treatment of vestibular schwannoma. Neuro-Oncology.

[31] Louis D.N., Perry A., Wesseling P., Brat D.J., Cree I.A., Figarella-Branger D., Hawkins C., Ng H.K., Pfister S.M., Reifenberger G. (2021). The 2021 WHO Classification of Tumors of the Central Nervous System: A summary. Neuro-Oncology.

[32] Samii M., Matthies C. (1997). Management of 1000 Vestibular Schwannomas (Acoustic Neuromas): Hearing Function in 1000 Tumor Resections. Neurosurgery.

[33] Hummel M., Perez J., Hagen R., Gelbrich G., Ernestus R.-I., Matthies C. (2016). When Does Hearing Loss Occur in Vestibular Schwannoma Surgery? Importance of Auditory Brainstem Response Changes in Early Postoperative Phase. World Neurosurg..

[34] Moffat D.A., Kasbekar A., Axon P.R., Lloyd S.K.W. (2012). Growth Characteristics of Vestibular Schwannomas. Otol. Neurotol..

[35] Breun M., Schwerdtfeger A., Martellotta D.D., Kessler A.F., Monoranu C.M., Matthies C., Hagemann C., Löhr M. (2020). ADAM9: A novel player in vestibular schwannoma pathogenesis. Oncol. Lett..

[36] Feldheim J., Kessler A.F., Schmitt D., Salvador E., Monoranu C.M., Feldheim J.J., Ernestus R.-I., Löhr M., Hagemann C. (2020). Ribosomal Protein S27/Metallopanstimulin-1 (RPS27) in Glioma—A New Disease Biomarker?. Cancers.

[37] Schindelin J., Arganda-Carreras I., Frise E., Kaynig V., Longair M., Pietzsch T., Preibisch S., Rueden C., Saalfeld S., Schmid B. (2012). Fiji: An open-source platform for biological-image analysis. Nat. Methods.

[38] Schneider C.A., Rasband W.S., Eliceiri K.W. (2012). NIH Image to ImageJ: 25 Years of image analysis. Nat. Methods.

[39] Feldheim J., Kessler A.F., Schmitt D., Wilczek L., Linsenmann T., Dahlmann M., Monoranu C.M., Ernestus R.-I., Hagemann C., Löhr M. (2018). Expression of activating transcription factor 5 (ATF5) is increased in astrocytomas of different WHO grades and correlates with survival of glioblastoma patients. OncoTargets Ther..

[40] Hanemann C.O., Bartelt-Kirbach B., Diebold R., Kämpchen K., Langmesser S., Utermark T. (2006). Differential gene expression between human schwannoma and control Schwann cells. Neuropathol. Appl. Neurobiol..

[41] Zhou L., Lyons-Rimmer J., Ammoun S., Müller J., Lasonder E., Sharma V., Ercolano E., Hilton D., Taiwo I., Barczyk M. (2016). The scaffold protein KSR1, a novel therapeutic target for the treatment of Merlin-deficient tumors. Oncogene.

[42] Cooper J., Giancotti F.G. (2015). Molecular insights into *NF2*/Merlin tumor suppressor function. FEBS Lett..

[43] Wang G., Fu Z., Li D. (2014). MACC1 overexpression and survival in solid tumors: A meta-analysis. Tumor Biol..

[44] Graamans K., Van Dijk J.E., Janssen L.W. (2003). Hearing deterioration in patients with a non-growing vestibular schwannoma. Acta Oto-Laryngol..

[45] Masuda A., Fisher L.M., Oppenheimer M.L., Iqbal Z., Slattery W.H., Natural History Consortium (2004). Hearing Changes after Diagnosis in Neurofibromatosis Type 2. Otol. Neurotol..

[46] Asthagiri A.R., Vasquez R.A., Butman J.A., Wu T., Morgan K., Brewer C.C., King K., Zalewski C., Kim H.J., Lonser R.R. (2012). Mechanisms of Hearing Loss in Neurofibromatosis Type 2. PLoS ONE.

[47] Sagers J.E., Sahin M.I., Moon I., Ahmed S.G., Stemmer-Rachamimov A., Brenner G.J., Stankovic K.M. (2019). NLRP3 inflammasome activation in human vestibular schwannoma: Implications for tumor-induced hearing loss. Hear. Res..

[48] Bommakanti K., Seist R., Kukutla P., Cetinbas M., Batts S., Sadreyev R.I., Stemmer-Rachamimov A., Brenner G.J., Stankovic K.M. (2023). Comparative Transcriptomic Analysis of Archival Human Vestibular Schwannoma Tissue from Patients with and without Tinnitus. J. Clin. Med..

[49] Franchi L., Eigenbrod T., Núñez G. (2009). Cutting Edge: TNF-α Mediates Sensitization to ATP and Silica via the NLRP3 Inflammasome in the Absence of Microbial Stimulation. J. Immunol..

[50] Kobelt D., Zhang C., Clayton-Lucey I.A., Glauben R., Voss C., Siegmund B., Stein U. (2020). Pro-inflammatory TNF-α and IFN-γ Promote Tumor Growth and Metastasis via Induction of MACC1. Front. Immunol..

[51] Breun M., Schwerdtfeger A., Martellotta D.D., Kessler A.F., Perez J.M., Monoranu C.M., Ernestus R.-I., Matthies C., Löhr M., Hagemann C. (2018). CXCR4: A new player in vestibular schwannoma pathogenesis. Oncotarget.

[52] Long J., Zhang Y., Huang X., Ren J., Zhong P., Wang B. (2021). A Review of Drug Therapy in Vestibular Schwannoma. Drug Des. Dev. Ther..

[53] Schulz A., Büttner R., Hagel C., Baader S.L., Kluwe L., Salamon J., Mautner V.-F., Mindos T., Parkinson D.B., Gehlhausen J.R. (2016). The importance of nerve microenvironment for schwannoma development. Acta Neuropathol..

[54] Nattmann A., Breun M., Monoranu C.M., Matthies C., Ernestus R.-I., Löhr M., Hagemann C. (2020). Analysis of ADAM9 regulation and function in vestibular schwannoma primary cells. BMC Res. Notes.

[55] Juneja M., Kobelt D., Walther W., Voss C., Smith J., Specker E., Neuenschwander M., Gohlke B.-O., Dahlmann M., Radetzki S. (2017). Statin and rottlerin small-molecule inhibitors restrict colon cancer progression and metastasis via MACC1. PLOS Biol..

[56] Gohlke B., Zincke F., Eckert A., Kobelt D., Preissner S., Liebeskind J.M., Gunkel N., Putzker K., Lewis J., Preissner S. (2022). Real-world evidence for preventive effects of statins on cancer incidence: A trans-Atlantic analysis. Clin. Transl. Med..

[57] Tran S., Killeen D.E., Qazi S., Balachandra S., Hunter J.B. (2021). Association of Metformin with the Growth of Vestibular Schwannomas. Otolaryngol. Head Neck Surg..

