# Supplementary material for: Metastasis Associated in Colorectal Cancer 1 (MACC1) mRNA Expression Is Enhanced in Sporadic Vestibular Schwannoma and Correlates to Deafness
Source: Cancers (Basel). 2023 Aug 14;15(16):4089. doi: 10.3390/cancers15164089 (PMC10452285; doi:10.3390/cancers15164089)
Supplement: Supplementary file 1 [file cancers-15-04089-s001.zip › Supplementary Figure 24.07.2023.pdf]

Supplementary Materials

Metastasis associated in colorectal cancer 1 (MACC1) mRNA expression is enhanced in sporadic vestibular schwannoma and correlates to deafness

Maria Breun, Katharina Flock, Jonas Feldheim, Anja Nattmann, Camelia M. Monoranu, Pia Herrmann, Ralf-Ingo Ernestus, Mario Löhr, Carsten Hagemann and Ulrike Stein

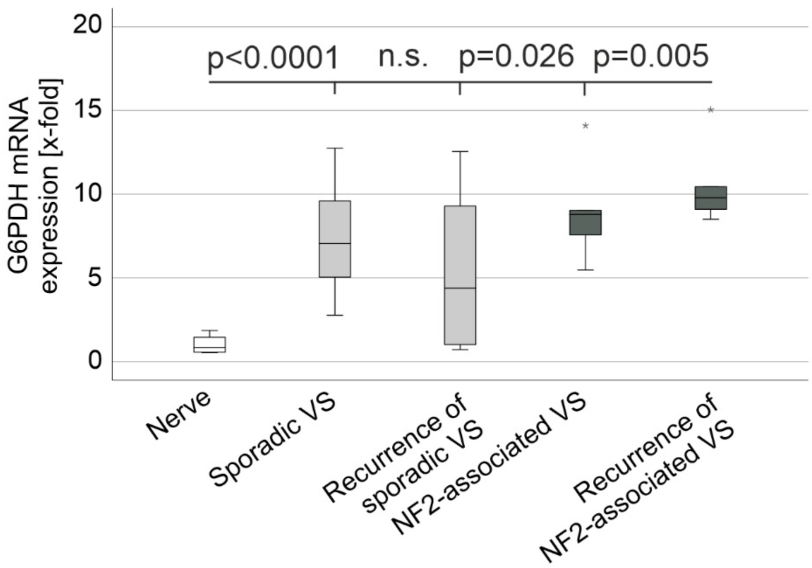

Figure S1. Box-plot of Glucose-6-phosphate dehydrogenase (G6PDH) mRNA expression in normal vestibular nerve tissue (nerve, n = 4) and vestibular schwannoma (VS) specimen (sporadic VS, n = 22; recurrence of sporadic VS, n = 5; *NF2*-associated VS, n = 5; recurrence of *NF2*-associated VS, n = 5). The p-values (ANOVA) refer to the nerve. The different VS-entities were not significantly different from each other, while G6PDH-expression in the normal vestibular nerve was reduced, probably due to increased disintegration in the autopsy material. Therefore, G6PDH was not suitable for comparison analyses of VS with normal vestibular nerve tissue, while it remained an appropriate housekeeping gene to compare VS entities with each other. n.s. = not significant.
